# Metatranscriptomic Analyses of Diel Metabolic Functions During a *Microcystis* Bloom in Western Lake Erie (United States)

**DOI:** 10.3389/fmicb.2019.02081

**Published:** 2019-09-10

**Authors:** Emily J. Davenport, Michelle J. Neudeck, Paul G. Matson, George S. Bullerjahn, Timothy W. Davis, Steven W. Wilhelm, Maddie K. Denney, Lauren E. Krausfeldt, Joshua M. A. Stough, Kevin A. Meyer, Gregory J. Dick, Thomas H. Johengen, Erika Lindquist, Susannah G. Tringe, Robert Michael L. McKay

**Affiliations:** ^1^Department of Biological Sciences, Bowling Green State University, Bowling Green, OH, United States; ^2^Cooperative Institute for Great Lakes Research (CIGLR), University of Michigan, Ann Arbor, MI, United States; ^3^Department of Microbiology, The University of Tennessee, Knoxville, Knoxville, TN, United States; ^4^Graduate School of Genome Science and Technology, The University of Tennessee, Knoxville, Knoxville, TN, United States; ^5^Department of Earth and Environmental Sciences, University of Michigan, Ann Arbor, MI, United States; ^6^U.S. Department of Energy Joint Genome Institute, Walnut Creek, CA, United States; ^7^Great Lakes Institute for Environmental Research, University of Windsor, Windsor, ON, Canada

**Keywords:** *Microcystis*, metatranscriptomics, microcystin, cyanobacterial blooms, Lake Erie

## Abstract

This study examined diel shifts in metabolic functions of *Microcystis* spp. during a 48-h Lagrangian survey of a toxin-producing cyanobacterial bloom in western Lake Erie in the aftermath of the 2014 Toledo Water Crisis. Transcripts mapped to the genomes of recently sequenced lower Great Lakes *Microcystis* isolates showed distinct patterns of gene expression between samples collected across day (10:00 h, 16:00 h) and night (22:00 h, 04:00 h). Daytime transcripts were enriched in functions related to Photosystem II (e.g., *psbA*), nitrogen and phosphate acquisition, cell division (*ftsHZ*), heat shock response (*dnaK*, *groEL*), and uptake of inorganic carbon (*rbc*, *bicA*). Genes transcribed during nighttime included those involved in phycobilisome protein synthesis and Photosystem I core subunits. Hierarchical clustering and principal component analysis (PCA) showed a tightly clustered group of nighttime expressed genes, whereas daytime transcripts were separated from each other over the 48-h duration. Lack of uniform clustering within the daytime transcripts suggested that the partitioning of gene expression in *Microcystis* is dependent on both circadian regulation and physicochemical changes within the environment.

## Introduction

Cyanobacterial harmful algal blooms (cHABs), dominated primarily by *Microcystis*, have recurred annually in the open waters of western Lake Erie since the mid-1990s (Brittain et al., 2000; Steffen M.M. et al., 2014) with blooms increasing in severity and duration over the past decade (Michalak et al., 2013; Bullerjahn et al., 2016). Within a bloom, a subset of strains of *Microcystis* spp. are capable of producing microcystins, which are known hepatotoxins and potential tumor promoters (Falconer, 1994; Fan et al., 2014). Consequently, within the western Lake Erie watershed, cHABs result in increased costs for water treatment and are responsible for economic declines related to tourism, property values, and recreational fisheries (Bingham et al., 2015; Wolf and Klaiber, 2017).

*Microcystis* spp. can dominate late summer phytoplankton communities due to a variety of adaptive strategies. Cells over-winter in the sediments where they can be recruited to surface waters during the summer as light availability and temperatures increase (Brunberg and Blomqvist, 2003; Rinta-Kanto et al., 2009; Kitchens et al., 2018). *Microcystis* can also promote and tolerate the formation of pH extremes that preclude the growth of competing eukaryotes (Krausfeldt et al., 2019). Buoyancy resulting from gas vesicles allows cells to control their position in the water column, thus shaping light and nutrient availability (Reynolds et al., 1987; Brookes and Ganf, 2001). The genomic architecture of *Microcystis aeruginosa* is thought to be “plastic” due to horizontal gene transfer as well as the activity of transposases and restriction modification enzymes encoded within its genome (Kaneko et al., 2007; Frangeul et al., 2008; Steffen M. et al., 2014; Meyer et al., 2017). These functions are presumed to generate genetic diversity within the cyanobacterial population via deletion, duplication and/or acquisition of genes from the endemic community into the genome. Together, these mechanisms offer adaptive strategies to maintain competitive dominance (Humbert et al., 2013). *Microcystis* spp. also possess a variety of genes and pathways to compete for light and nutrients, including uptake systems for various nitrogen and carbon species (Valladares et al., 2002; Badger and Price, 2003). Increasing temperatures are also favorable for growth of *M. aeruginosa*, whose optimum growth temperature (>25°C) is typically higher than that of other phytoplankton species (Reynolds, 2006; Jöhnk et al., 2008). In many lakes, increasing temperatures consistent with climate change will likely strengthen vertical stratification thereby reducing mixing and allowing phytoplankton growth at the surface to remain undisturbed, promoting formation of surface blooms (Huisman and Hulot, 2005; Paerl and Huisman, 2008).

A critical adaptation of bloom-forming phytoplankton is the regulation of processes according to diel light availability. Circadian oscillators are genetic regulators of expression operating at a period of about 24 h. The circadian “clock” functions as a regulator that anticipates daily environmental changes that can shape cell metabolism. Circadian rhythms function as a constant, entrained by cycles of light/dark, independent of temperature effects (Bünning, 1973; Pittendrigh, 1981; Johnson and Hastings, 1986; Sweeney, 2013). These functions were initially observed in eukaryotes and thought to occur only within *Eukarya* (Konopka and Benzer, 1971), until their discovery in cyanobacteria (Kondo et al., 1993; Lorne et al., 2000). The *kaiABC* gene cluster and its physiological outputs within cyanobacteria have been shown to specifically control the rhythmicity of cell functions (Ishiura et al., 1998; reviewed in Welkie et al., 2019). The photosynthetic nature of cyanobacteria presumes the circadian pacemaker will initiate expression of some genes to anticipate dawn to maximize daytime functions such as photosynthetic light harvesting. Other physiological functions have been shown to yield maximum expression at subjective midday (Kucho et al., 2005). The cyanobacteria clock controls global gene expression by regulating the activity of all promoters (Liu et al., 1995; Xu et al., 2003; Labiosa et al., 2006).

Multiple cellular functions within cyanobacteria are coupled to circadian rhythms, including nutrient acquisition and assimilation, amino acid uptake, respiration, carbohydrate synthesis, replication, and cell division (Chen et al., 1991; Kramer et al., 1996; Golden et al., 1997; Ishiura et al., 1998). While fluctuating environmental conditions (light, temperature, pH, nutrient availability) may invoke stress responses, it is important to understand the mechanisms and range of circadian control that may mask or overlay expression resulting from transient stress. Indeed, patterns of gene expression under changing conditions of light, temperature and nutrient starvation are distinct from those under global circadian control. With respect to cHAB events, it is important to differentiate these two patterns (Labiosa et al., 2006; Penn et al., 2014). Additionally, diel patterns of expression can exist in cyanobacteria in the absence of a circadian clock (Holtzendorff et al., 2008). Whereas this study alone cannot sort out what functions are regulated by KaiABC, this work, along with future studies, can begin to understand the interplay of environmental cues and the circadian pacemaker.

This study queried expression of key metabolic functions to understand the ecophysiology of a *Microcystis* spp. bloom over the course of diel cycles. Specifically, a metatranscriptomic approach was undertaken to study temporal changes in the metabolic functions of an August 2014 *Microcystis* spp.-dominated bloom. Just 3 weeks prior, this event resulted in a “do not drink” order issued for Toledo, OH due to detection of microcystins in the finished water supply above the 1 μg L^–1^ World Health Organization (WHO) drinking water advisory (Bullerjahn et al., 2016; Steffen et al., 2017). Metatranscriptome analyses paired with environmental metadata provided insight into factors related to bloom success and toxicity, along with a better understanding of bloom metabolism throughout the day and night, particularly with regard to photosynthesis, nutrient assimilation and microcystin production. Overall, this information can help inform the development of new strategies toward prediction of bloom toxigenicity and mitigation of bloom events.

## Materials and Methods

### Sample Collection

**FIGURE 1:**
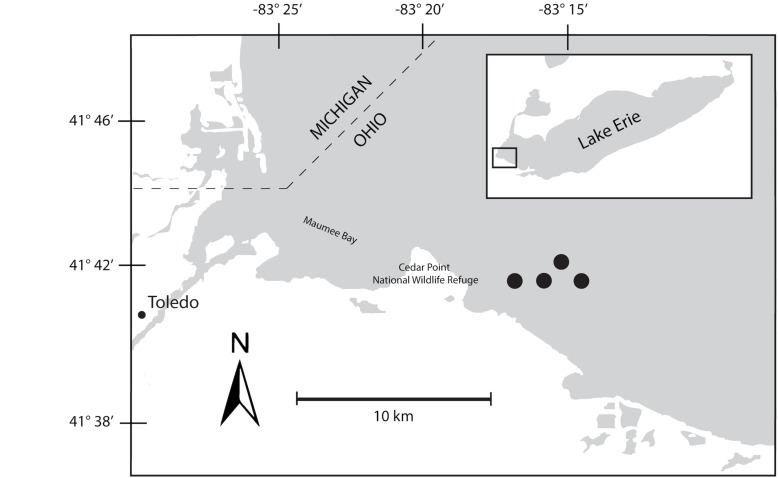
Map of Maumee Bay, western basin of Lake Erie indicating movements of bloom during 48-h survey. The seven sampling events were mapped to the four sites indicated on the map.

A 48-h Lagrangian survey of the 2014 *Microcystis* bloom was conducted in western Lake Erie in late August 2014. Our study was designed to track the bloom over diel cycles using a drifter with GPS capabilities deployed near the Toledo water intake. Over the course of the survey, the drifter moved roughly 2 km through water depths varying from 10–15 m (Figure 1). Samples were collected at 6-h intervals beginning on August 26, at 22:00 h, producing two sets of triplicate samples from 22:00 h (samples 1S, 5S), one set of triplicates at 04:00 h (2S), and two sets of triplicates at 10:00 h (3S, 6S), and 16:00 h (4S, 7S). A sample for 04:00 h over the second diel cycle was not collected due to adverse weather that precluded sampling. At each sampling time point, triplicate water samples for chlorophyll and nutrients were collected adjacent to the drifter by hand casting a Niskin bottle to a depth of 1 m. Biomass from each water sample was collected onto Sterivex cartridge filters (0.22 μm; EMD Millipore, Billerica, MA, United States) using a peristaltic pump. Filters were immediately stored in liquid nitrogen upon field collection followed by transfer to −80°C until RNA extraction.

### Physico-Chemical Measurements

Chlorophyll-*a* (chl *a*) biomass was measured by concentrating lake water on a glass fiber filter (GF/F, 47 mm diameter, Whatman, Maidstone, United Kingdom) at low vacuum pressure under low light conditions and stored at −20°C until extraction. Samples were extracted with N, N-dimethylformamide and analyzed by fluorescence with a 10AU fluorometer (Turner Designs, Sunnyvale, CA, United States; Speziale et al., 1984).

Phycocyanin concentrations were measured by concentrating lake seston on a glass fiber filter (GF/F, 47 mm diameter, Whatman). Sodium phosphate buffer (pH 6.8; Ricca Chemical, Batesville, IN, United States) was added to the filter and phycocyanin was extracted using two freeze-thaw cycles followed by sonication. Relative fluorescence was measured using a Turner AquaFluor (Sunnyvale, CA, United States) and converted to phycocyanin concentration using a series of dilutions of a commercial standard (Sigma-Aldrich, St. Louis, MO, United States; Horváth et al., 2013). For total phosphorus (TP), duplicate 50 mL aliquots of whole lake water were collected into acid-washed glass culture tubes and stored at 4°C until analysis within 1 week. For dissolved nutrients, duplicate whole water samples were collected in a triple rinsed (ultrapure water) 20 mL syringe and filtered through 0.22 μm nominal pore-size nylon filters into 15 mL collection tubes that were stored at −20°C until analysis. Dissolved inorganic nitrogen (DIN) and phosphate concentrations were determined using standard automated colorimetric procedures as modified by Davis and Simmons (1979) on a QuAAtro Continuous Flow Analyzer (SEAL Analytical, Inc., Mequon, WI, United States) according to methods detailed by the manufacturer and in compliance with United States. EPA Methods 365.4, 350.1, and 353.1. TP and total dissolved phosphorus (TDP) used the same analysis following a persulfate digestion adapted from Menzel and Corwin (1965). Particulate microcystins were measured by filtering whole lake water onto a 3-μm pore-size polycarbonate membrane and kept at −20°C until analysis. Particulate microcystins were extracted from samples using a combination of physical and chemical lysis techniques. All samples were resuspended in 1 mL molecular grade water (pH 7; Sigma-Aldrich) and subjected to three freeze-thaw cycles before the addition of the QuikLyse reagents (Abraxis LLC, Warminster, PA, United States) per the manufacturer’s instructions. The samples were then centrifuged for 5 min at 2 × 10^3^ *g* to pellet cellular debris. The concentrations of microcystins (reported as microcystin-LR equivalents) were measured using a microcystin enzyme-linked immunosorbent assay (Abraxis LLC) following methods standardized by the manufacturer (Fischer et al., 2001). This assay is congener-independent and detects the ADDA moiety, a shared moiety among microcystins. The detection limit of the assay was 0.04 μg L^–1^.

### Nucleic Acid Extraction and Sequencing

RNA was extracted from a single Sterivex cartridge from each sampling time using the PowerWater DNA Isolation Kit for Sterivex (Qiagen, Carlsbad, CA, United States), modified for RNA using manufacturer’s protocols. To improve RNA yield, Sterivex^®^ cartridges were vortexed for 5 min longer than recommended each time and all wash buffers were allowed to sit for 1 min before vacuum extraction through the binding column. DNase treatment was performed as recommended in the protocol using the On-Spin Column DNase kit (QIAGEN). This protocol was optimized by allowing the DNase solution to sit for an extra 15 min than recommended. RNA was checked for DNA contamination by PCR with universal 16S primers (27F and 1522R). Any additional DNase treatments required were performed using the Turbo DNase kit (Ambion, Austin, TX, United States). rRNA was removed from 1 μg of total RNA using Ribo-Zero rRNA Removal Kit (Epicenter, Madison, WI, United States). Stranded cDNA libraries were generated using the TruSeq Stranded Total RNA LT kit (Illumina, Inc., San Diego, CA, United States). The rRNA depleted RNA was fragmented and reversed transcribed using random hexamers and Superscript II reverse transcriptase (Invitrogen, Carlsbad, CA, United States) followed by second strand synthesis. The fragmented cDNA was treated with end-pair, A-tailing, adapter ligation, and eight cycles of PCR. The prepared libraries were quantified using a KAPA Library Quantification kit (Kapa Biosystems, Wilmington, MA, United States) and run on a LightCycler 480 real-time PCR instrument (Roche Diagnostics Corp., Indianapolis, IN, United States). The quantified libraries were then multiplexed with other libraries, and the pool of libraries was then prepared for sequencing on the Illumina HiSeq sequencing platform utilizing a HiSeq Cluster kit, v4 (Illumina^TM^), and Illumina’s cBot instrument to generate a clustered flow cell for sequencing. Sequencing of the flow cell was performed on the Illumina HiSeq2500 sequencer using a TruSeq SBS sequencing kit, v4, following a 2 × 150 indexed run recipe (Mavromatis et al., 2009). Metatranscriptomes obtained were accessed and downloaded through the Integrated Microbial Genomes platform (IMG) developed by U.S. DOE Joint Genome Institute (JGI) (Markowitz et al., 2012, 2014) and the JGI genome portals (Nordberg et al., 2014). Raw unassembled metagenomic sequence data were uploaded to the online server MG-RAST (Meyer et al., 2008) for assembly attribute data, phylogenetic, and functional analysis.

### Bioinformatics and Statistical Analysis

Analyses and visualization of data were performed using CLC Genomics Workbench v 12.0.2 (Qiagen CLC Bio). Sequences were imported utilizing the Illumina High-Throughput Sequencing Import function. Low-quality reads and failed reads were automatically removed. The reads were trimmed with a quality limit of 0.05 and an ambiguous base limit of 2. Automatic read-through adapter trimming was performed. RNA-Seq Analysis was performed using the raw reads of the seven diel transcriptomes against the following genomes: *M. aeruginosa* LE3 from Lake Erie (Brittain et al., 2000; Meyer et al., 2017), *Synechococcus elongatus* PCC 6301, *Sulfurimonas denitrificans* DSM 1251, *Desulfovibrio magneticus* RS-1, *Anabaena cylindrica* PCC 7122, *Aphanizomenon flos-aquae* NIES-81, *Klebsiella pneumoniae* 1158, and *Burkholderia pseudomallei* K96243, and an annotated genome of *Planktothrix agardhii* from Lake Erie obtained from Greg Dick at the University of Michigan. RNASeq parameters were: one reference sequence per transcript, mismatch cost of 2, insertion cost of 3, deletion cost of 3, length fraction of 0.8, similarity fraction of 0.8. Data output of expression values were calculated as Transcripts Per Million mapped reads (TPM) through RNA-Seq function to normalize within each sample and manually normalized across all samples with a ratio to the housekeeping gene *gyrB* TPM.

Principal Component Analysis (PCA) was performed using the CLC Genomics Workbench (Qiagen CLC Bio) to assess relationships between diel samples with regards to expression. TPM gene expression plots were created in R 3.5.1 (R Core Team, 2018) using the packages tidyr 0.8.2 (Wickham and Henry, 2018) and ggplot2 v3.1.0 (Wickham, 2016). Raw sequences are available from the NCBI sequence read archive under SRP117911, SRP117914, SRP117915, SRP117922, SRP128942, SRP128945, and SRP128954.

## Results

### Survey Physico-Chemical Properties

**FIGURE 2:**
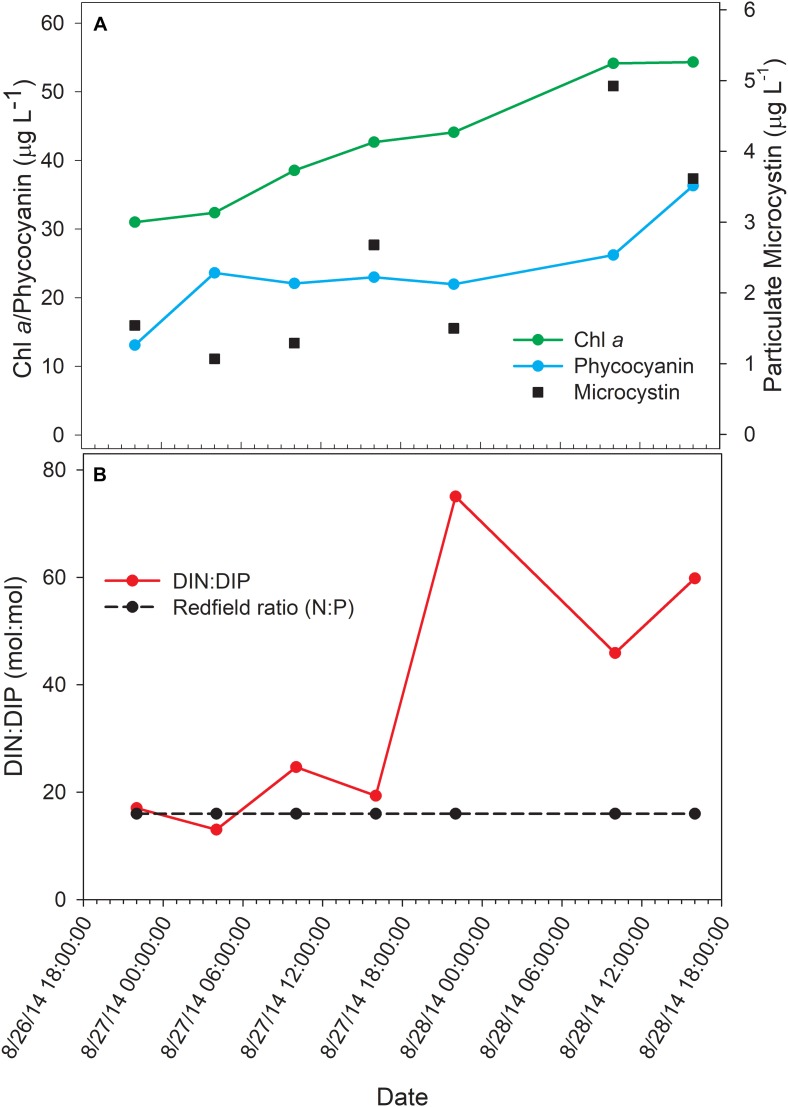
Physico-chemical water data over the course of the 48-h Lagrangian study. **(A)** Photopigment and microcystin toxin concentrations; **(B)** dissolved inorganic nitrogen and phosphorus ratios.

The bloom tracked a southwesterly course traveling nearly 2 km over the 48-h survey in late August (Figure 1). Whereas winds originated from S/SW leading up to buoy deployment and initial bloom tracking, wind direction switched in the early morning of August 27 and remained E/NE with daily averages of 5–6 knots for the remainder of the survey (weather data from KPCW: Erie-Ottawa International Airport; KTDZ: Toledo Executive Airport). Western Lake Erie is shallow (*Z*_*mean*_ = 7.4 m) and characterized as polymictic. Whereas buoyancy afforded by gas vesicles helped to maintain *Microcystis* colonies near the surface, wind gusts up to nine knots over the course of the survey likely promoted mixing as was predicted using a Lagrangian particle tracking model (Rowe et al., 2016) applied to the same geographic area preceding the early August 2014 cHAB at the Toledo water intake (Steffen et al., 2017). Increases in chl *a* biomass of 75% and more than a doubling of phycocyanin (PC) indicated that cyanobacterial biomass was increasing over the survey (Figure 2). Molar ratios of dissolved inorganic nitrogen to dissolved inorganic phosphorus (DIN:DIP) showed only minor variation around Redfield stoichiometry (N:P = 16:1; dashed line) during the first 18 h of sampling (Figure 2). The system then shifted to P-deficiency (N:P ∼ 40–60) during day 2 of the survey. Particulate microcystins, were initially between 1 and 2 μg L^–1^, but reached as high as 5 μg L^–1^ toward the end of the survey (Figure 2). Archived weather reports from KPCW and KTDZ confirmed skies to be clear leading up to the start of the survey and switching to variable cloud cover during the morning hours of day 2^1^. Irradiance both days were similar (Supplementary Figure S1), yielding maximum PAR at the nearby Toledo intake crib of 1542 μmol quanta m^–2^s^–1^ on August 27 and 1573 μmol quanta m^–2^s^–1^ on August 28^2^.

### Phylogenetic Classification of Transcripts

Seven metatranscriptomes were produced from the Western Lake Erie water samples (1.97 Gbp assembled) with read counts ranging from 109,124 to 570,660 per sample. rRNA accounted for ∼1% of the assembled metatranscriptomes. Fifty seven percent of the mRNA was annotated as encoding known proteins, the remaining ∼40% encoded unknown proteins. Of the predicted proteins, ∼70% were assigned to functional categories. Less than 5% of reads (per transcriptome) failed quality control tests.

**FIGURE 3:**
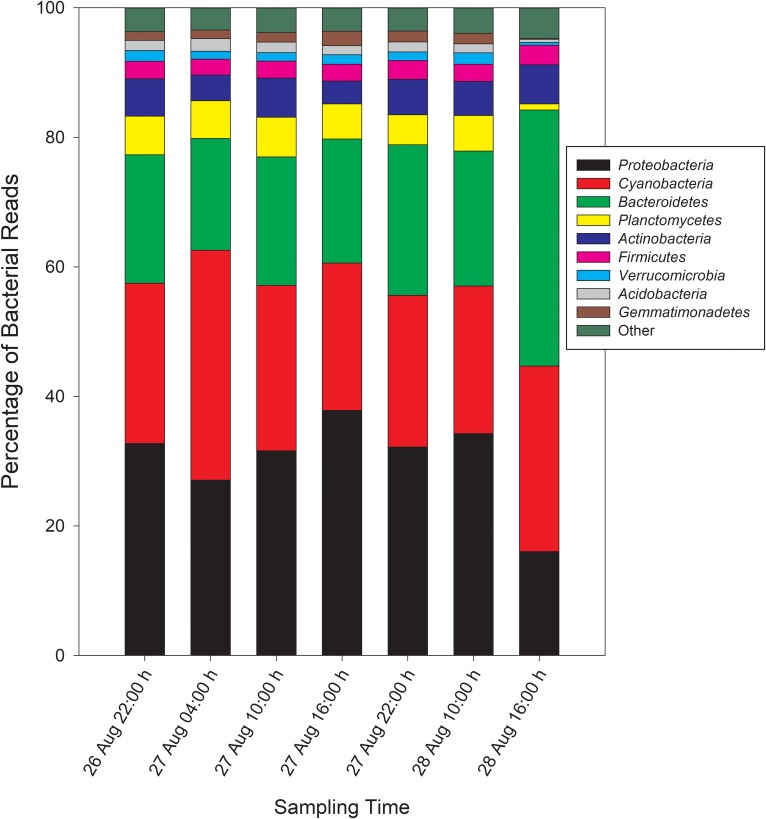
Phylogenetic breakdown of transcripts over the course of the 48-h sampling period – community composition by phyla. “Other” refers to bacterial reads that could not be unambiguously assigned to a phylum.

**FIGURE 4:**
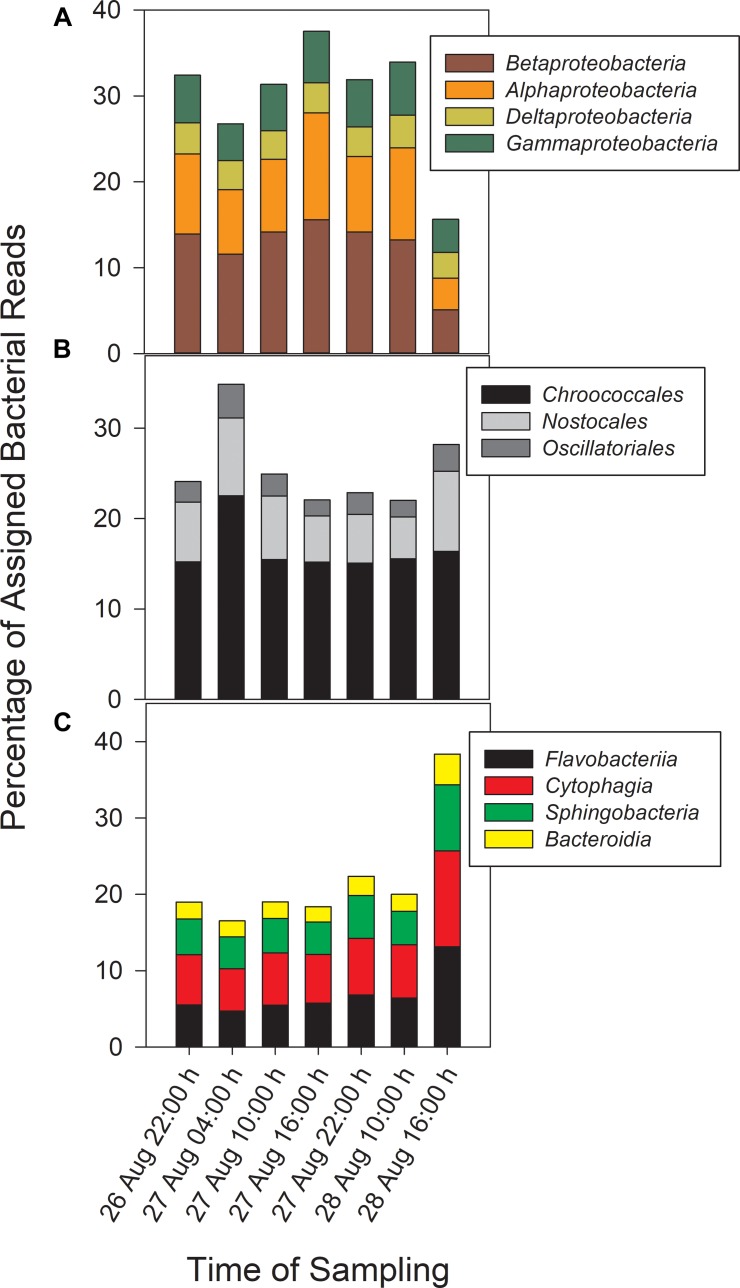
Abundances of *Proteobacteria* **(A)** and *Cyanobacteria* **(B)** reads by order and *Bacteroidetes* reads **(C)** by class.

Taxonomic analysis derived from MG-RAST showed reads dominated by *Bacteria* (∼70% of all reads) and *Eukaryota* (∼30%). *Archaea* and viral reads represented <1% of all transcripts. Of the *Bacteria* reads, *Cyanobacteria* (22–35%), *Proteobacteria* (16–37%), and *Bacteroidetes* (17–40%) were prominently represented in each metatranscriptome (Figure 3). The *Bacteroidetes* were dominated by the classes *Cytophagia* (∼30% of *Bacteroidetes* reads), *Flavobacteria* (∼30%), and *Sphingobacteria* (∼25%), whereas β-*Proteobacteria* were the most abundant proteobacterial reads (∼40%) followed by α-*Proteobacteria* (∼30%), γ-*Proteobacteria* (∼15%), and δ-*Proteobacteria* (Figure 4). Of the *Cyanobacteria*, ∼65% were order *Chroococcales*. *Microcystis* was the dominant genus, contributing half of the *Chroococcales* population. Classes *Nostocales* and *Oscillatoriales* contributed one-third of the *Cyanobacteria*, namely genera *Nostoc* and *Dolichospermum* within the *Nostocales*.

### Differential Relative Abundance of *Microcystis* spp. Transcripts

**FIGURE 5:**
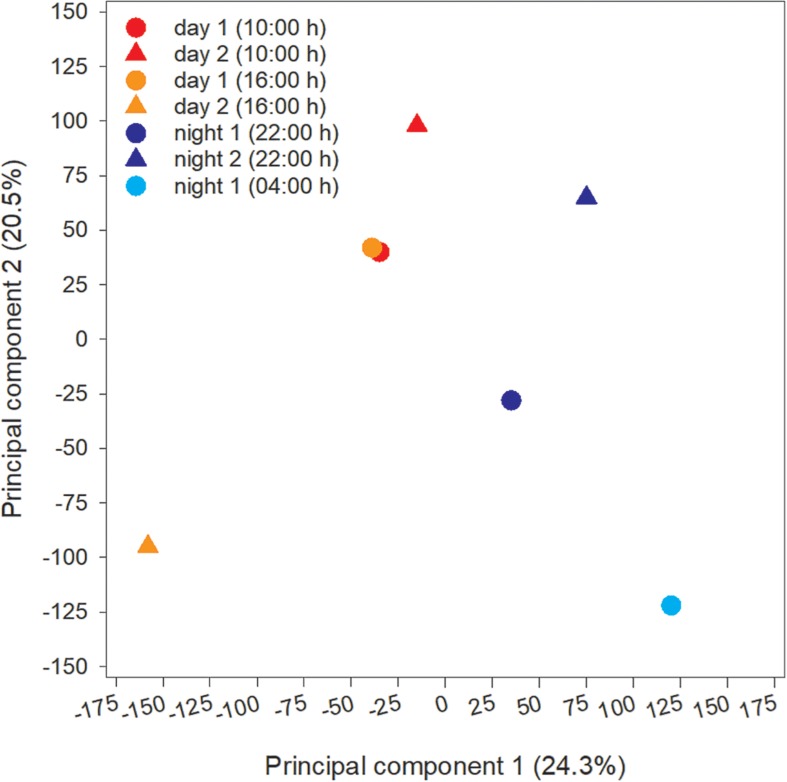
Principal Component Analysis (PCA) of transcripts obtained from each time point during the study.

The microcystin toxin-producing *Microcystis* LE3 genome isolated from Lake Erie was recruited to annotate metatranscriptomes to increase transcript coverage compared to publicly available *Microcystis* genomes currently available in the National Center for Biotechnology Information (GenBank assembly accession numbers GCA_000010625.1, GCA_000981785.2, GCA_001704955.2, GCA_002095975.1). PCA of differential expression and transcript abundance partitioned the seven transcriptomes into two general day and night groups (Figure 5). Variability within the day and night sample groups likely reflect day-to-day and hour-by-hour changes in nutrient and light availability, resulting in changes in gene expression patterns.

### Highly Transcribed Genes

Supplementary Table S1 summarizes the most transcripts at each time point. In all samples, tmRNA transcripts were uniformly the most abundant, indicating active mechanisms were in place ensuring translational fidelity (Takada et al., 2002). Whereas many of the highly transcribed genes encoded gene products of unknown function, gas vesicle genes (*gvp*) were highly transcribed at all time points, and high light inducible (*hli*) and *psbA* transcripts were abundant by day, reflecting their role in Photoprotection, turnover and repair of Photosystem II (Kulkarni and Golden, 1994; Hutin et al., 2003). Genes encoding Hsp20 were highly transcribed by day and phycobilisome transcripts (*cpcAB*) were abundant in night samples.

### Photosynthesis – Light Reactions

**FIGURE 6:**
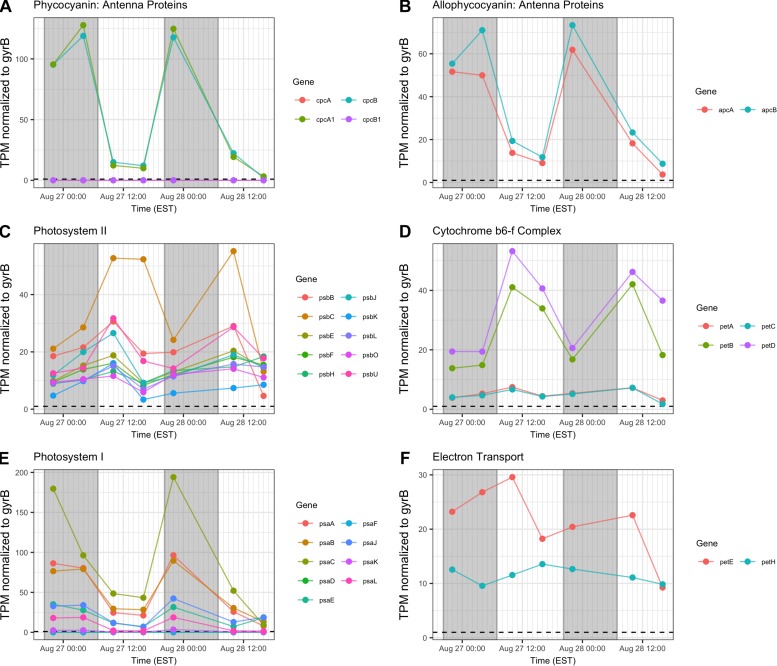
Diel transcriptional patterns of *Microcystis* photosynthesis genes. **(A,B)** Phycobilisome subunits; **(C)** Photosystem II; **(D)** cytochrome *b_6_/f*; **(E)**, Photosystem I; **(F)**, ferredoxin and plastocyanin. Gray shading indicates time periods between sunset and sunrise.

Gene transcripts associated with antenna function, Photosystems I and II (PSI, PSII) and the cytochrome *b_6_/f* complex were assessed and revealed that relative abundance of phycobilisome (*cpc, apc*) and PSI (*psaA-L*) transcripts compared to *gyrB* increased primarily at night (Figures 6A,B,E). Conversely, PSII (*psb*), *b_6_/f* complex (*petA-D*), ferredoxin and plastocyanin (*petEFH*) transcript abundance increased by day, yielding maximum relative abundance at 10:00 h (Figures 6C,D,F). *psbA*, encoding the PSII D1 protein, was analyzed separately due to its very high relative daytime expression due to high rates of DI protein turnover (Supplementary Table S1), and *psbA* expression matched the daytime pattern of other PSII-associated genes.

### Photosynthesis – Ci Assimilation

**FIGURE 7:**
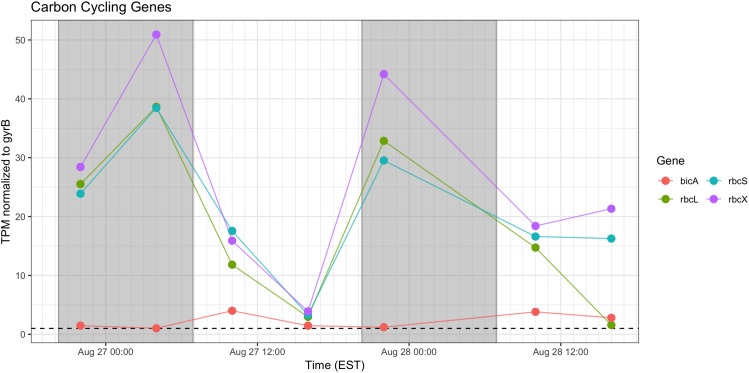
Diel transcriptional patterns of *Microcystis* inorganic carbon acquisition genes. Gray shading indicates time periods between sunset and sunrise.

Genes associated with Rubisco (*rbcLSX*) showed higher relative transcript abundance at night (04:00 h) during the first day of sampling and a pronounced minimum by the next afternoon (16:00 h), but during Day 2, the pattern was less distinct, as relative transcript abundance proceeded throughout the daytime hours (Figure 7). *bicA* transcripts were detected at low levels during the day, but with an inconsistent pattern from Day 1 to Day 2 that may reflect variation in bicarbonate availability.

### Nutrient Acquisition

**FIGURE 8:**
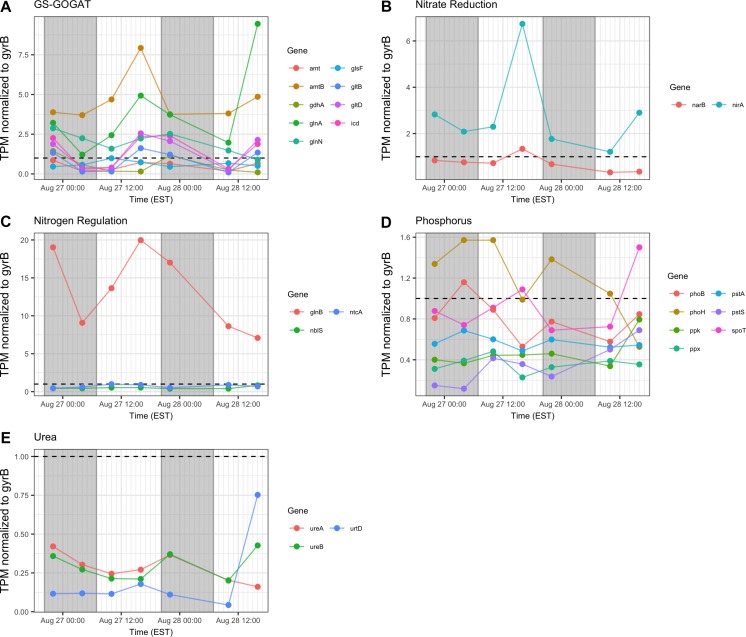
Transcriptional patterns of *Microcystis* nutrient assimilation genes. **(A)** GS-GOGAT pathway; **(B)** nitrite reductase; **(C)** N regulation genes; **(D)** phosphorus assimilation; **(E)** urea assimilation. Gray shading indicates time periods between sunset and sunrise.

In general, transcription of nutrient acquisition functions was more active during the daytime hours. Genes encoding functions associated with N acquisition and N-responsive gene regulation yielded different patterns of transcript abundance. Whereas transcripts associated with the GS-GOGAT pathway and ammonium uptake (*amt, glnAN, glsE, gltBD, icd*) had a peak relative abundance in the afternoon (16:00 h), urea transporters and urease showed a more variable pattern and comparatively low relative expression throughout the two day period (Figures 8A,C,E). Relative transcript abundance for genes encoding nitrate and nitrite reduction also followed a daytime pattern, peaking at 16:00 h on Day 1 (Figure 8B). By contrast, transcripts detected associated with phosphorus acquisition and uptake (*pho, pst*) exhibited no clear diel pattern (Figure 8D).

### Cell Division

**FIGURE 9:**
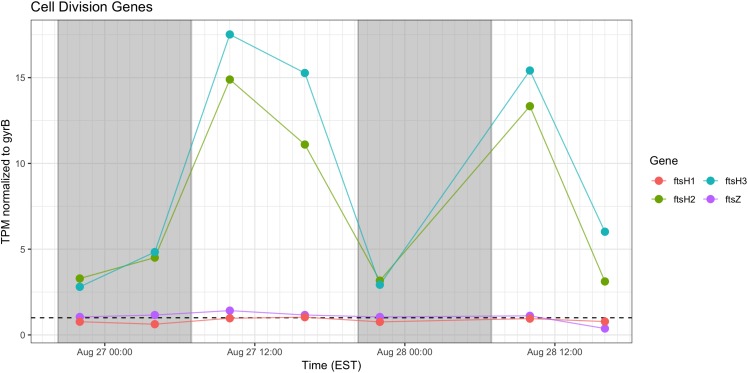
Transcriptional patterns of *Microcystis* cell division genes *ftsH* and *ftsZ*. Gray shading indicates time periods between sunset and sunrise.

Relative abundance of transcripts encoding the septation ring protein FtsZ and cell division function FtsH peak during the day on both days, indicating that cell division likely followed later during the daytime hours (Figure 9). Proxy measurements for bloom biomass suggested that the bloom was expanding at the time of the sampling (Figure 2), and the pattern of *fts* transcription reflects active growth of *Microcystis* during this time.

### Microcystin Synthesis and Stress Responses

**FIGURE 10:**
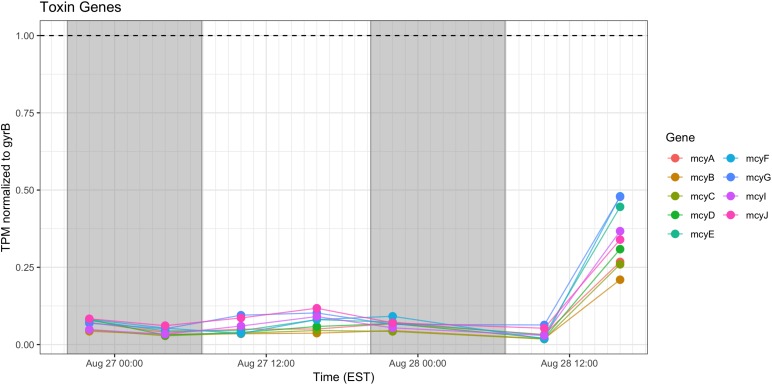
Transcriptional patterns of *Microcystis* microcystin biosynthesis genes. Gray shading indicates time periods between sunset and sunrise.

Relative expression of the *mcy* genes was very low and was elevated only during the afternoon of Day 2. Microcystin measured during the course of the sampling show a pattern of increased toxin, trending toward highest concentrations during morning of the second day (Figure 10).

**FIGURE 11:**
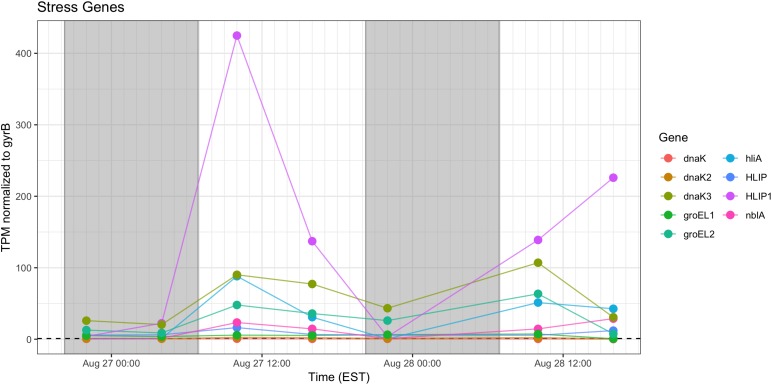
Transcriptional patterns of *Microcystis* stress response genes (heat shock, high light, phycobilisome stability). Gray shading indicates time periods between sunset and sunrise.

Stress response genes examined included those involved in heat shock (*groEL, dnaK*), phycobilisome stability (*nblA*) and high light stress (*hliA*). Unsurprisingly, given increased daytime temperature, photosynthetic oxygen production and daytime irradiance, all genes showed increased relative transcription at 10:00 h and 16:00 h (Figure 11).

## Discussion

Metatranscriptomes spanning two diel cycles were produced from a toxic cyanobacterial bloom in western Lake Erie during late August 2014. The survey described here coincided with a mid-bloom phase with the bloom persisting through October (Steffen et al., 2017). A Lagrangian approach was adopted to ensure that a common patch of biomass was sampled over the 48-h experiment. In this way, changes in relative abundance of transcripts could be attributed to either diel patterns or changing physico-chemical conditions but not to sampling of different bloom populations. Taxonomic analysis of recruited transcripts showed *Microcystis* spp. to dominate cyanobacterial reads during the survey consistent with independent surveys of the western basin conducted during August 2014 (Berry et al., 2017; Steffen et al., 2017).

**FIGURE 12:**
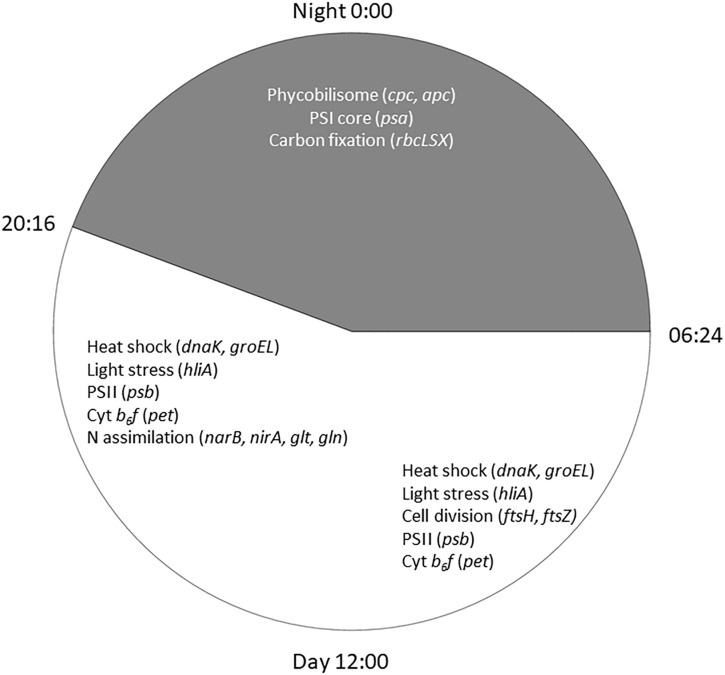
Summary of significant *Microcystis* functions expressed during the 24 h cycle in the Toledo, 2014 bloom event. Lines of the clock indicate sunrise at 0654 h and sunset at 2016 h on August 26, 2014.

Cyanobacteria are known to regulate gene expression under circadian influences (Golden et al., 1997). The intent of this study was to identify cellular processes within a *Microcystis* bloom that show diel patterns of expression. PCA of *Microcystis* transcriptomes revealed grouping of samples based upon time of collection (i.e., “day” and “night”). Variability amongst day samples as shown by PCA (Figure 5) suggested circadian rhythms were not the sole determiner of expression, rather transient environmental changes may have contributed to differing transcript accumulation. A summary of transcriptional activity throughout the diel cycle is presented in Figure 12.

Reflecting the demands of a phototrophic lifestyle, photosynthesis-related functions for the dark and light reactions were detected at all time points. Photosynthesis functions appear to partition so that PSII-dependent O_2_ evolution peaks in mid-afternoon, as has been observed in laboratory studies (Garczarek et al., 2001; Mackenzie and Morse, 2011). Expression of genes involved in the cyt *b_6_/f* complex follow this same pattern. However, PSI genes were preferentially expressed at night, peaking at 22:00 h. Given the variability of the timing of PSI gene expression in N-fixing cyanobacteria (del Carmen Muñoz-Marin et al., 2019), and that degradation of PSI occurs during the night in *Crocosphaera watsonii* WH8501 (Masuda et al., 2018), nighttime expression of *psa* genes in *Microcystis* may be a genus-specific trait.

Consistent with modest nitrogen depletion during the first 18 h as revealed by nutrient stoichiometry, expression of nitrogen assimilation genes was detected, as has been reported previously for western Lake Erie (Harke and Gobler, 2013, 2015; Steffen M. et al., 2014; Steffen et al., 2017). In the present survey, expression of nitrogen metabolism genes may reflect both transient nitrogen availability as well as circadian control. Indeed, elevated transcript abundance for *glnA* at the conclusion of sampling on Day 2 (16:00 h) may suggest environmental stresses resulting in reduced N bioavailability (Figure 8). However, the increasing DIN/DIP ratio over the course of the survey indicated a greater demand for P. Transcripts related to nitrogen assimilation that accumulated during the day included *narB* and *nirA* (encoding nitrate and nitrite reductase) and *glnA* (encoding glutamine synthetase), indicating active mechanisms of nitrogen assimilation and synthesis of amino acids in daylight (Mérida et al., 1991; Kramer et al., 1996; Marzluf, 1997; Wyman, 1999; Flores and Herrero, 2005). Overall, nitrogen metabolism transcripts exhibited increases in relative abundances during the light period. Since nitrate reduction is an ATP-dependent process, nitrate assimilation may be regulated by day so that it is temporally aligned with daytime photosynthetic energy generation.

Regarding phosphate uptake, Penn et al. (2014) described highest transcription of alkaline phosphatase (*pho*) and phosphate transported (*pst*) genes during the day in a *Microcystis* bloom event, in this study, no such pattern is seen. Such differences may also be due to transient changes in P availability during the course of each survey. Indeed, analysis of dissolved nutrients over the course of the survey revealed a pronounced shift in dissolved N:P ratio (Figure 2).

Cyanobacteria are known to contain multiple inorganic carbon uptake genes and pathways, facilitating variable responses to availability of carbon for photoautotrophic growth (Sandrini et al., 2014, 2015). A growing concern in bloom formation and mitigation is the response of cHAB species to increasing atmospheric CO_2_ (Verspagen et al., 2014; Visser et al., 2016). Whereas C fixation genes were preferentially expressed at night, carbon concentrating mechanism transcripts did not appear to express distinct diel patterns (data not shown). The relative transcript abundance of Rubisco genes in the predawn hours of Day 1 and elevated expression throughout Day 2 is in partial agreement with Wyman (1999) examining *rbcL* expression in natural populations of marine *Synechococcus*, and Straub et al. (2011), who documented a decline in *rbc* expression during daytime in culture experiments with *M. aeruginosa* PCC 7806. The data in this study are in some agreement with the findings of Sandrini et al. (2016) who demonstrated similar variable diel responses during a bloom event, in which *bicA* transcription was also correlated to bicarbonate concentration.

As expected, given environmental changes through the day, a diel pattern exists regarding transcript abundance for genes encoding stress and cell division proteins. *Synechococcus* cells have shown a specific gating of cell division independent of the circadian clock in avoidance of peak irradiance exposure (Mori et al., 1996). In agreement with this observation, relative transcript abundance for the ATP-binding *ftsH* and septation gene *ftsZ* increased during the morning, suggesting cell division at midday or in the afternoon (Figure 9). Relative abundance of the heat shock chaperones *dnaK*, *groEL* and high-light inducible gene *hliA* all peak during the day (Figure 11), similar to previous reports (Aoki et al., 1995).

Microcystin biosynthesis gene (*mcy*) transcript levels were very low (Figure 10), although transcripts were modestly higher during Day 2. Other studies have indicated toxin production to occur early in the night in a tropical bloom event (Penn et al., 2014) or as a daytime function in a culture experiment (Straub et al., 2011). Further complicating the issue is the observation that microcystin production is elevated at lower temperatures (Peng et al., 2018). Furthermore, previous studies that have examined the western Lake Erie *Microcystis* blooms have shown that toxin concentrations increase and *mcy* genes are significantly upregulated when the ambient communities are experimentally exposed to elevated nitrogen concentrations and high light, especially during August and September (Chaffin et al., 2018). Due to the low level of expression, it remains impossible within our data set to separate diel and light-driven effects on toxin production from the subtle effects of minor temperature fluctuations on both toxin gene transcription and toxin biosynthesis (Peng et al., 2018). However, what is known is that changes in toxic to non-toxic *Microcystis* strain ratios can be significantly influenced by physiochemical parameters such as nutrients and temperature (Davis et al., 2009, 2010) and that the shift in the toxic: non-toxic ratio will likely lead to changes in bloom toxin concentrations (O’Neil et al., 2012; Gobler et al., 2016). As such, both changes in gene expression and *Microcystis* community composition need to be taken into account when determining the relationship between the toxin concentration and the molecular underpinnings of production.

Examining the relative transcript abundance for the genes analyzed in the study reveals a marked increase in relative transcript abundance for a few genes (e.g., *rbcS, rbcX, glnA, mcyA-J*) at the final time point (16:00 h on Day 2). This increase likely indicated a change in environmental conditions on the second day. The nutrient profile shifted toward P deficiency on the 28th (Figure 2), suggesting the onset of modest nutrient stress, but light stress was likely not a factor given the similar peak irradiances measured at the Toledo water intake crib during both days (Supplementary Figure S1). This documented change in gene expression remains unexplained, but since the sample passed QA/QC at JGI it was not due to changes in transcript composition due to rRNA contamination or RNA degradation (data not shown). High abundance of viral reads were also not detected.

## Conclusion

Lake Erie experiences annual cHAB events, and these events will continue as eutrophication intensifies (Watson et al., 1997; Michalak et al., 2013). In light of concerns over the safety of water resources and human health, there is an urgent need to elucidate the factors influencing cHAB formation, proliferation, and maintenance, to better inform prevention and mitigation strategies. Toward this goal, the present study used a metatranscriptomic approach to investigate metabolic function of a Lake Erie *Microcystis* bloom over diel cycles. Lab studies have found the genomes of *Microcystis* to be highly plastic and adaptable to the environment, increasing their competitive ability (Meyer et al., 2017). Previous investigations report the presence of circadian regulation of gene expression in cyanobacteria (Kondo et al., 1993; Golden et al., 1997). The circadian clock has been shown to enhance the fitness of the species within the microbial community. Paired with a highly adaptive genome, *Microcystis* has the potential to be very successful. Our analysis indicates that Lake Erie *Microcystis* likely utilizes efficient organization of gene expression to maintain productivity, such as utilization of a variety of nutrient species throughout a diel cycle (CO_2_, bicarbonate, nitrate, ammonium), cell division and, if a toxic genotype, the production of microcystins. Our results suggest that although diel patterns are detectable, environmental cues also influence regulation, as supported by PCA analysis. The next step of this analysis is to study the metabolism of the global microbial community. In doing so, those results paired with this analysis of *Microcystis* spp. will provide insights into factors leading to the natural mitigation of a bloom, and how the surrounding consortium of heterotrophs and phages interact and influence both cHAB success and decline. Indeed, a role for *Microcystis* phage infection was suggested in constraining this same bloom 3 weeks earlier in Lake Erie, yielding shifts in microcystin toxin from an intracellular particulate fraction to the soluble phase (Steffen et al., 2017). Conversely, recent work invokes the Black Queen Hypothesis (Morris et al., 2012) in demonstrating a role for catalase produced by bloom-associated heterotrophs in protecting *Microcystis* from oxidative stress thus promoting bloom success (Greg Dick, personal communication).

## Data Availability

The datasets generated for this study can be accessed from NCBI SRA, SRP117911, SRP117914, SRP117915, SRP117922, SRP128942, SRP128945, and SRP128954.

## Author Contributions

ED conducted the field sampling, initial analysis of the assembled reads, and wrote the Introduction and Materials and Methods sections. MN and PM analyzed the relative transcript abundance at each diel timepoint. MD, LK, and JS isolated RNA and processed the metatrascriptomic data from JGI. KM, GD, TD, SW, and RM developed the Lagrangian sampling plan. GB helped to devise the study, analyzed the metadata, and wrote the Results and Discussion sections. TJ led the field sampling and provided all field metadata. ST and EL processed the RNAs for sequencing.

## Conflict of Interest Statement

The authors declare that the research was conducted in the absence of any commercial or financial relationships that could be construed as a potential conflict of interest.

## Footnotes

1 https://flightaware.com/live/airport/KPCW/weather

2 http://lees.geo.msu.edu/research/sensor_net.html
