# Supplementary figures and images for: Metatranscriptomic Analyses of Diel Metabolic Functions During a Microcystis Bloom in Western Lake Erie (United States)
Source: Front Microbiol. 2019 Sep 10;10:2081. doi: 10.3389/fmicb.2019.02081 (PMC6746948; doi:10.3389/fmicb.2019.02081)

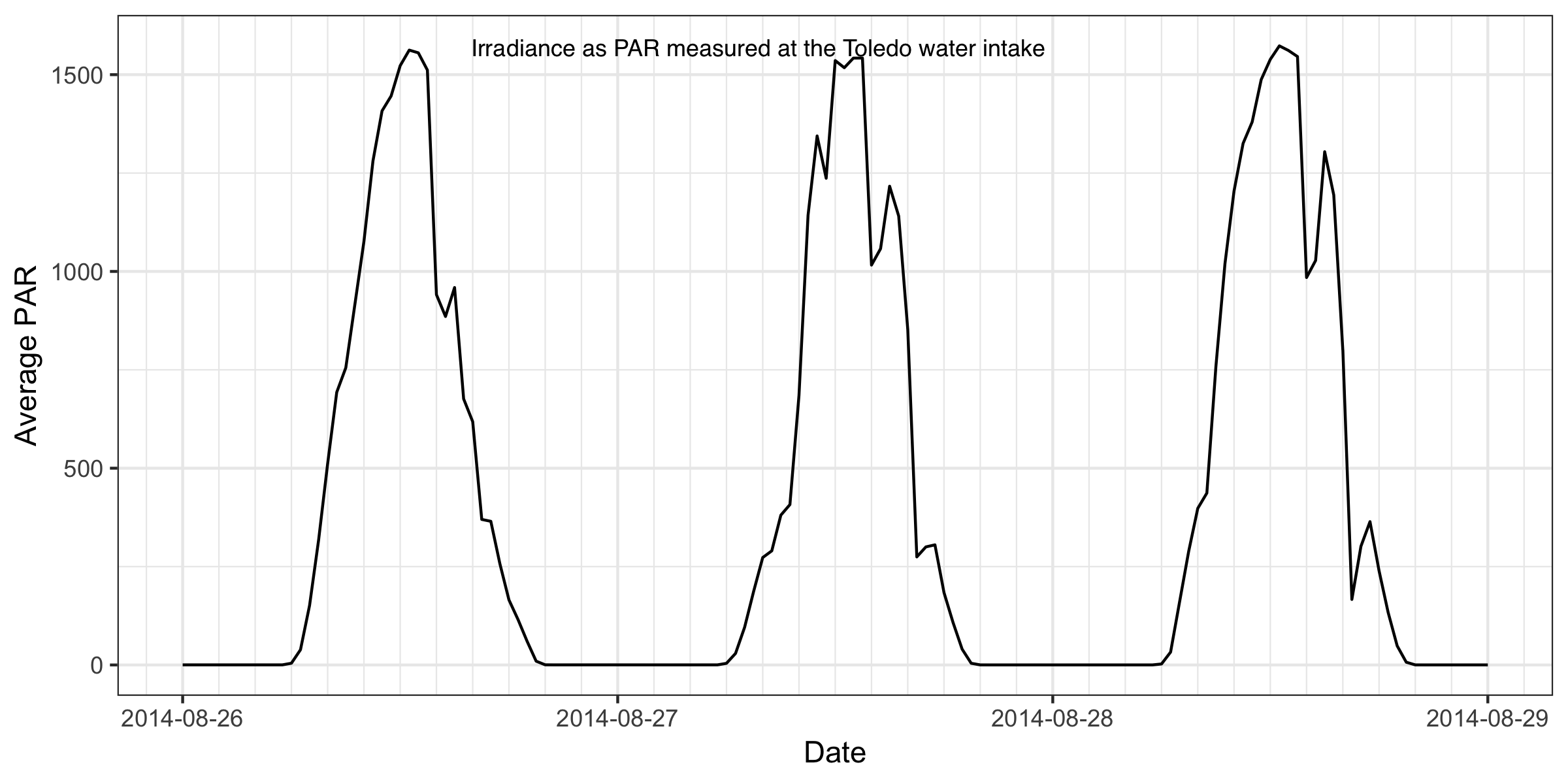

Supplement: Supplementary file 1 [file Image_1.png]
